# How to interpret right ventricular remodeling in athletes

**DOI:** 10.1002/clc.23350

**Published:** 2020-03-04

**Authors:** María Sanz‐de la Garza, Amelia Carro, Stefano Caselli

**Affiliations:** ^1^ Cardiovascular Institute, Hospital Clínic Barcelona Spain; ^2^ Corvilud Institute Asturias Spain; ^3^ Cardiovascular Center Zürich, Klinik im Park Zürich Switzerland

**Keywords:** arrhythmogenic cardiomyopathy, athlete, endurance training, right ventricle

## Abstract

Long‐lasting athletic training induces an overload on the heart that leads to structural, functional, and electrical adaptive changes known as the “athlete's heart.” The amount of this heart remodeling has been traditionally considered balanced between the left and the right heart chambers. However, during intense exercise, the right heart is exposed to a disproportional afterload and wall stress which over a long period of time could lead to more pronounced exercise‐induced changes. Highly trained athletes, especially those involved in endurance sport disciplines, can develop marked right ventricular (RV) remodeling that could raise the suspicion of an underlying RV pathology including arrhythmogenic cardiomyopathy (ACM). The distinction between physiological and pathological RV remodeling is essential as ACM is a common cause of sudden cardiac death in athletes, and high‐intensity exercise training has demonstrated to accelerate its phenotypic expression and worsen its prognosis. The distinction between physiological and pathological RV remodeling is essential since ACM is a common cause of sudden cardiac death in athletes, and high‐intensity exercise training has demonstrated to accelerate the phenotypic expression and worsen the prognosis. This article outlines the physiological adaptation of the RV to acute exercise, the subsequent physiological structural and functional changes induced by athletic training and provides useful tips of how to differentiate between physiological RV remodeling and a cardiomyopathy phenotype.

## 1 RIGHT VENTRICULAR AND PULMONARY CIRCULATION ADAPTATIONS TO EXERCISE

**Figure 1:**
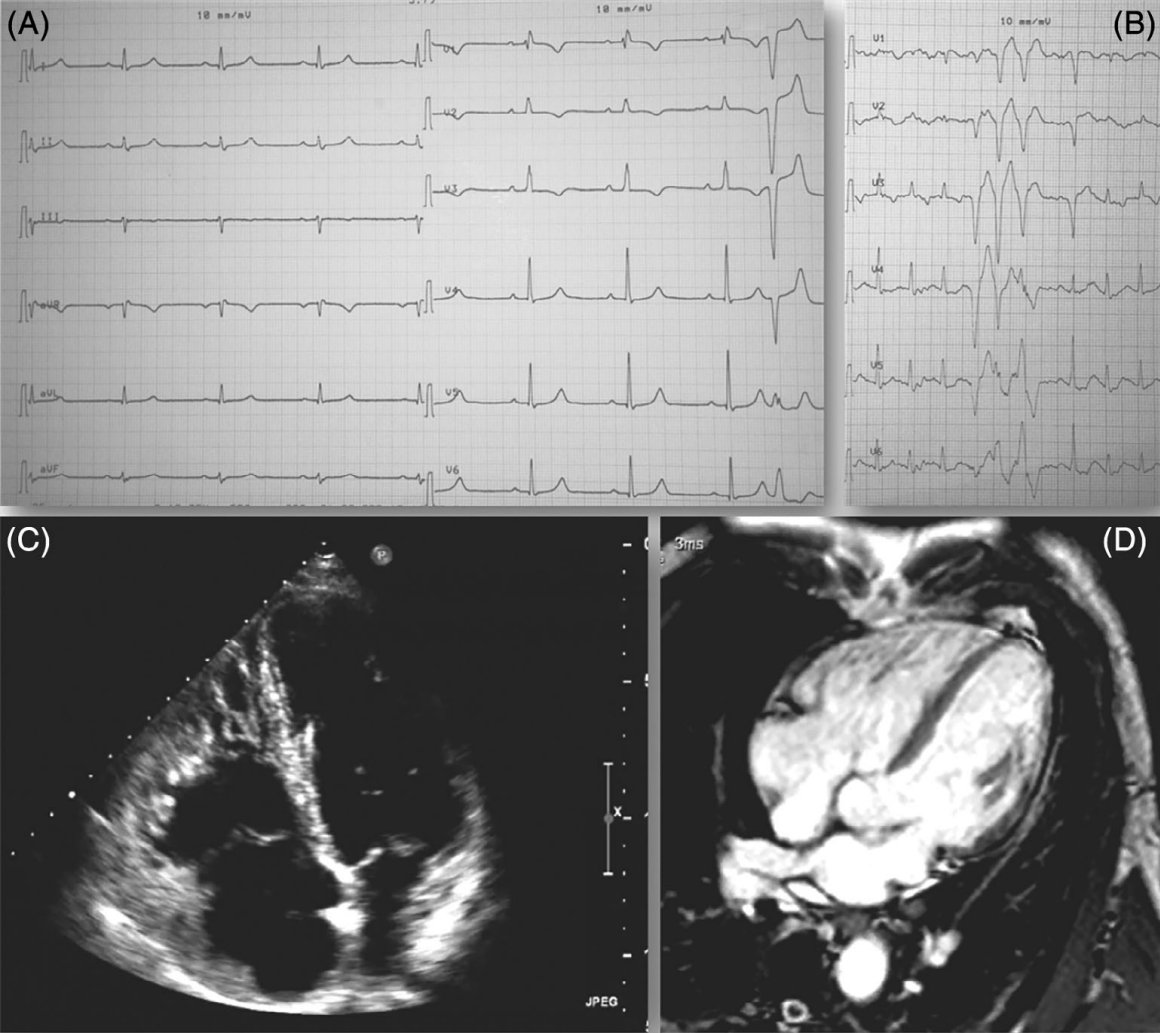
Marked right ventricular remodeling in a veteran endurance athlete. The figure shows an example of extreme right ventricular remodeling rising the suspicion of arrhythmogenic cardiomyopathy. This is a 61 years old male competitive cyclist with more than 40 years of uninterrupted high intensity endurance training. He was asymptomatic and with negative family history for cardiomyopathies or sudden cardiac death. On resting ECG, A, incomplete right bundle branch block together with negative T waves in V1‐V3 was observed. Exercise Testing, B, showed a non‐sustained ventricular tachycardia of three beats with LBBB and inferior axis (from the RVOT). Echocardiography, C, revealed a significantly dilated right ventricle with huge trabeculations. The RVOT in short axis measured 18 mm/m^2^ and in long axis 22 mm/m^2^. The RV basal diameter was 58 mm. The global systolic function of the RV was normal with a fractional area change of 44% and with no evidence of regional wall motion abnormalities. Cardiac Magnetic Resonance, D, confirmed marked dilatation without dysfunction of the RV. The RV/LV volume ratio was 1.2. No areas of LGE were identified on the left and right ventricles. Despite the presence of ECG changes, arrhythmias, and significant RV dilatation there were no sufficient criteria for a diagnosis of arrhythmogenic cardiomyopathy; specifically, the athlete was asymptomatic, had negative family history and imaging revealed absence of RV global or regional systolic dysfunction. A periodical follow‐up every 6 months was, however, advised

Under resting conditions, pulmonary circulation is characterized by high compliance, low vascular resistance, and low afterload. In contrast, the systemic circulation works with moderate compliance, higher vascular resistance, and higher afterload. During exercise, both the pulmonary and systemic circulations have to accommodate the same increase in cardiac output (CO) which can be 3 to 5 times higher with high‐intensity exercise and even rise to 40 L/min in highly trained athletes.^1^ This considerable increase in flow is well accommodated by the systemic circulation via an extensive dilation of the large vasculature, resulting in a marked reduction of vascular resistance and thus, a moderate increase in systemic pressure. However, the already low‐resistance, high‐compliance pulmonary vasculature has limited capacity to decrease vascular resistance and consequently requires higher increases in pulmonary artery pressure (PAP) in order to maintain the increased CO during exercise. Indeed, a linear relationship between the increase in CO and PAP has been shown in several studies.^1^ As such, the right ventricular (RV) is subjected to a greater load and greater wall stress than the left ventricle during exercise.^1^ In healthy individuals, the RV has enough contractile reserve to adapt to the rise in PAP during short‐term high‐intensity exercise.^2^ However, when high‐intensity exercise is maintained for several hours, RV function may be affected. Indeed, after an intense and prolonged endurance exercise, some studies have reported a reduction in RV systolic function,^3^ an increase in RV size^3^ and a rise in cardiac biomarkers,^4^, ^5^ suggesting a potential RV fatigue. This worsening in RV seems, however, to be transient, reversing within few days in healthy subjects. It has been postulated that repeated bouts of extreme endurance exercise may induce a phenotype similar to arrhythmogenic cardiomyopathy (ACM) with extreme dilatation of the RV, borderline or mildly reduced ejection fraction, and ventricular arrhythmias.^6^, ^7^ Figure 1 shows an illustrative example of an extreme electrical, structural, and functional remodeling in a veteran endurance athlete which may rise the suspicion of ACM. However, the hypothesis of an exercise‐induced cardiomyopathy has not been confirmed by other studies. Specifically, Aengevaeren et al^8^ assessed a cohort of 50 Olympic athletes of different sport disciplines participating to three consecutive Olympic games and reported only mild RV size changes over time and no deterioration of RV function. Bohm et al^9^ evaluated 33 elite master endurance athletes by MRI and 2D echocardiography, and reported no reduced indexes of RV systolic function and no pathological RV fibrosis. Nevertheless, there is growing evidence that RV adaptation are somewhat “individual related” and differs widely among subjects performing similar amounts of exercise.^10^ The mechanisms underlying this differential adaptation have not yet been clearly identified, but it may depend on a complex interplay of environmental and polygenetic factors also including different pulmonary vascular adaptations.^11^ Accurate identification of individuals at risk of life‐threatening arrhythmias and distinction between physiological and pathological RV remodeling is one of the main challenges for clinicians.

## 2 CLINICAL EVALUATION

Understanding the athlete's history is one of the strongest factors that either increases or decreases the likelihood of disease. RV remodeling in athletes should be interpreted in the context of each athlete training characteristics.^12^ The first history element that should be acknowledged is the type of sport and level of training.

Recently, a new classification of sport disciplines has been proposed, based on the relative isotonic and isometric components and subsequent cardiovascular adaptations: Skill disciplines (such as golf or horse‐riding), Power disciplines (such as weight‐lifting or short distance running), mixed disciplines (which include the team sports like soccer or basketball), and endurance disciplines (such as marathon, cycling, or cross‐country skiing).^13^ The highest degree of RV adaptations is usually observed in disciplines with a high isotonic component such as mixed or endurance disciplines.^14^

Endurance sports disciplines, weekly training load, years of training, and male gender have been identified as predictors of greater RV remodeling.^15^, ^16^

A family history of unexplained or unexpected sudden cardiac death before the age of 50, sudden infant death syndrome, drowning or near‐drowning, or unexplained car accident should raise the suspicion^16^ of a genetic arrhythmogenic disorder. It should be noted that close relatives include parents, grandparents, direct aunts/uncles, and siblings.^17^

The occurrence of prodromal symptoms or signs (ie, exertional chest pain, effort or recurrent syncope or pre‐syncope, palpitations or irregular heartbeats, exercise dyspnoea, or unexplained seizure) should prompt further diagnostic investigations. Some athletes, however, display nonspecific symptoms (ie, lethargy) and are regarded as asymptomatic; high level of suspicion and deep history taking is needed.^18^

## 3 ASSESSMENT OF THE ECG

**Figure 2:**
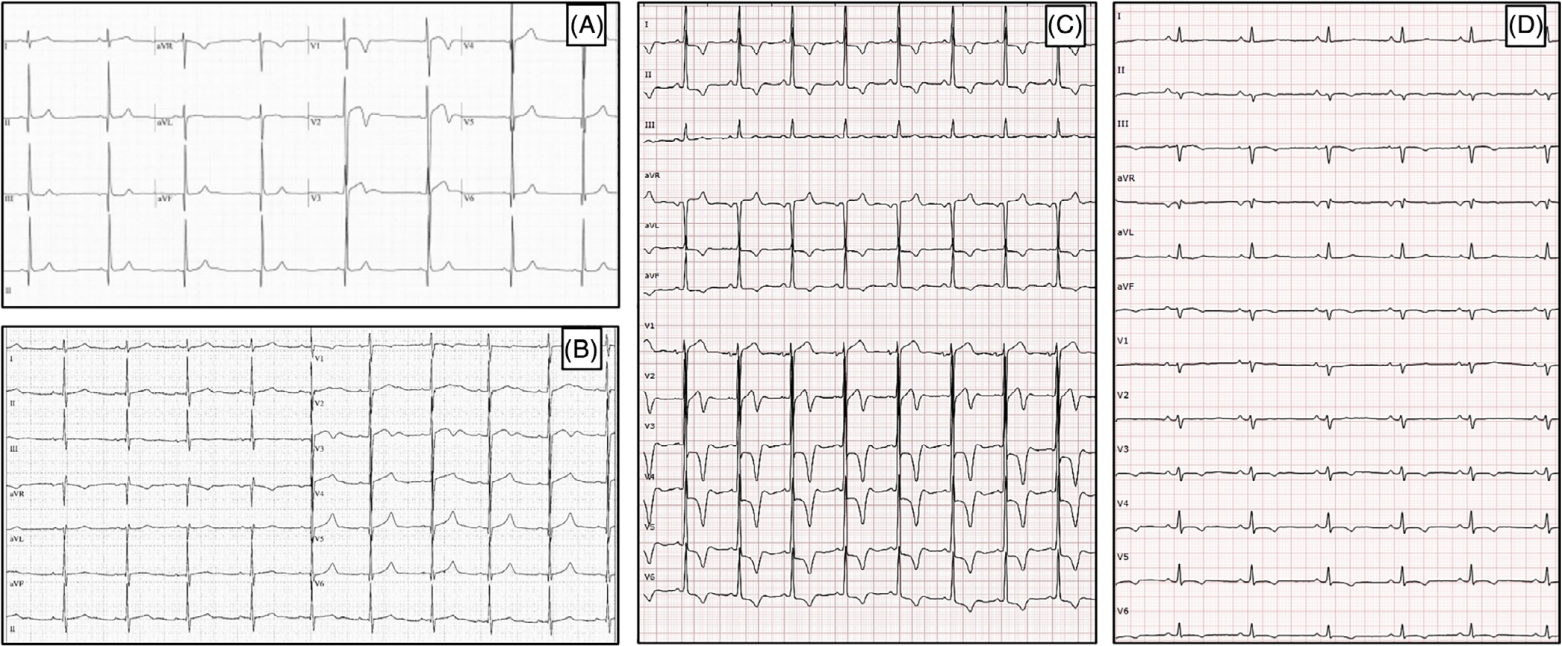
Normal and abnormal patterns of T‐wave inversion in athletes. A, T‐wave inversion in V1 to V3 preceded by J‐point elevation and convex ST‐segment elevation in a 24‐year‐old asymptomatic mixed race (Caucasian/American black) athlete without family history of sudden cardiac death. This should be considered a normal repolarization pattern in mixed black and Caucasian adult athletes. B, Anterior T‐wave inversion in V1 to V2 and biphasic T in V3 in a 15‐year‐old asymptomatic athlete. This a normal “juvenile” pattern. C, Abnormal T‐wave inversion in leads II, aVF, aVL, and V1 to V6, in a 30‐year‐old amateur endurance athlete with confirmed diagnosis of apical hypertrophic cardiomyopathy. D, Abnormal T‐wave inversion in V1 to V6, II and aVF preceded by isoelectric ST segments together with left axis deviation and small QRS complexes in a 28‐year‐old trail runner with confirmed diagnosis of arrhythmogenic cardiomyopathy with biventricular involvement

The ECG is one of the most important tools for the screening of athletes and for the identification of inherited cardiac conditions at risk of sudden cardiac death. A recently published international panel of experts released the new criteria for ECG interpretation in athletes. A correct definition of which ECG changes are physiological (common and training‐related ECG abnormalities) and which are pathological (uncommon and training‐unrelated ECG abnormalities) is essential for an accurate cardiovascular evaluation, preventing from false positive diagnosis, and facilitating cost savings.^19^ T wave‐inversion (TWI) in precordial leads (V1‐V3) and beyond in the absence of complete right bundle‐brunch block (RBBB) is major diagnostic criteria for ACM based on the 2010 Task Force Criteria (TFC) document.^20^ However, anterior TWI can occur in adolescent athletes (<16 years; “juvenile pattern of repolarization”) and it should be noted that is more common in women than in men^21^ (4.3% vs 1.4%, respectively; *P* < .001). In addition, in specific cases, anterior TWI may also be a manifestation of the physiological electrical changes induced by the exercise stimulus as it is present in up to 7% of endurance Caucasian athletes,^22^ and in up to 13% of African/afro‐Caribbean descents (black).^23^ In athletes, anterior TWI are commonly accompanied by other athlete's ECG features such as sinus bradycardia, J‐point elevation, or voltage criterion for left ventricular hypertrophy. In accordance with the 2017 International ECG recommendations for the interpretation of the athlete's ECG, the presence of anterior TWI can be considered as normal repolarization pattern in the athletes <16 years when limited to V1‐V3 and biphasic T wave in only V3 and in postpubertal black athletes when is confined to V1‐V4 and is preceded by J‐point elevation and convex ST segment as a normal repolarization pattern in black athletes.^19^ Figure 2 depicts normal (2A and 2B) and abnormal (2C and 2D) repolarization patterns in athletes.

Voltage criteria for RV hypertrophy and incomplete RBBB are common ECG findings in athletes. Zaidi et al^24^ studied the prevalence of the Sokolow‐Lyon voltage criteria for RV hypertrophy (R wave in lead V1 + S wave in lead V5 or V6 > 1.05 mV) in normal athletes, sedentary controls, and patients affected by ACM or pulmonary hypertension. The prevalence of RV hypertrophy was relatively in high healthy athletes and it did not correlate with RV wall thickness. In addition, none of the patients with ACM or pulmonary hypertension presented the voltage criteria for RV hypertrophy in isolation. In agreement with these findings, voltage criteria for RV hypertrophy in isolation is currently considered as another electrical feature of the athlete's ECG, representative of the normal spectrum of the training‐induced electrical adaptations. Complete RBBB has also been reported in up to 3% of athletes and correlates with RV size, being present in athletes with the greatest RV size.^25^

## 4 CARDIAC IMAGING

### 4.1 Echocardiography

Cardiovascular imaging should be performed after careful understanding of the athlete's characteristics. To rule out ACM in athletes, an accurate assessment of RV dimensional and functional parameters should be performed including the use of focused RV views.^26^ Many of the measured parameters can be affected by volume load and recent high‐intensity training, and hence, it is appropriate to ensure that the athlete is well hydrated and refrains from exercise for at least 6 hours prior to the examination.^27^

The athletic training promotes a global dilation of the RV cavity, with proportional increases in RV outflow (RVOT) and inflow. Up to 28% of athletes fulfill the RVOT dimensional major criteria for ACM, but the ratio RV outflow/inflow is unchanged with respect to the general population.^28^ This RV enlargement is known to be proportional to exercise training load and as such, elite athletes show the most dilated RV cavities, especially if involved in endurance sports.^15^, ^29^ Women, independent of training status, exhibit lower RV dimensions as compared to men^30^ and height and weight are known to influence all heart cavity dimensions. As such, athlete's body surface area and sex should be considered when evaluating exercise‐induced RV remodeling.^12^ Recent European recommendations suggest that only RV indexed measurements and major dimensional TFC should be used for defining a real enlargement of the RV, specifically an RVOT tract >19 mm/m^2^ in long axis view and >21 mm/m^2^ in short axis view are reported as cutoff value.^12^ Athletic training promotes a harmonic enlargement of the four heart cavities and as such, the exercise‐induced cardiac remodeling is expected to be balanced between the left and the right heart chambers. In contrast, right‐dominant ACM phenotypes are characterized by a disproportional dilation of the RV. A ratio of RV inflow dimension (in the apical view) and LV end‐diastolic dimension (parasternal long‐axis view) greater than 0.9 has been proposed as a cutoff to differentiate pathological from physiological RV remodeling in the athlete's population.^12^

**Figure 3:**
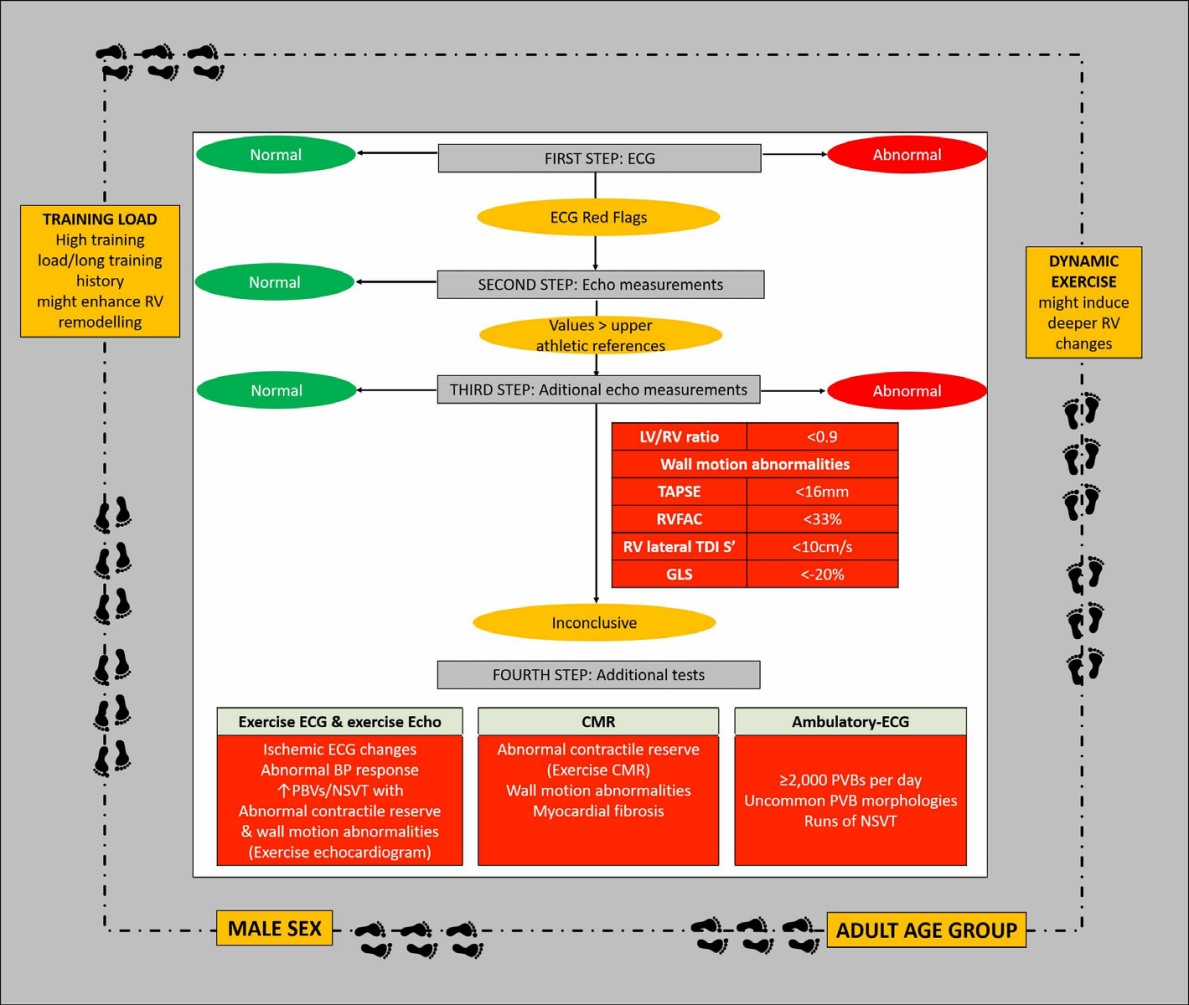
Proposed work‐flow in the differential diagnosis of physiological or pathological right ventricular remodeling. In each step, if parameters are within normal limits (green charts), no further exploration is required. If abnormal values are obtained (red charts), physiological remodeling is excluded and specific work‐up for underlying disease assessment should be prompted. FIRST STEP: electrocardiographic signs of abnormal RV remodeling include T‐wave inversion and repolarization patterns of abnormal RV adaptation to exercise (RED FLAGS) (for additional details, see the text). SECOND STEP: RV echo measurements. RV dimensions that exceed reference values for male/female athletes must be considered warning signs of abnormal RV remodeling (red flags); additional echo measurements (third step) should be assessed. THIRD STEP: additional echo measurements. Thresholds for abnormal RV remodeling are given based on several parameters (red flags).If any of these values is met, RV remodeling should not be considered physiologic (underlying disease). FOURTH STEP: Additional tests: Exercise ECG/exercise echo/cardiac magnetic resonance/ambulatory ECG monitoring are indicated if aforementioned resting examinations still inconclusive. The exercise ECG evaluates blood pressure response to exercise and can detect ischemia or arrhythmias induced by the exercise stimulus. Both exercise echocardiography and exercise CMR can assess the presence of exercise‐induced wall motion abnormalities and/or impaired contractile reserve. In addition, the presence of myocardial fibrosis can be unmasked by CMR late gadolinium enhancement. Arrhythmic burden can be assessed by an ambulatory ECG. The athlete's epidemiological characteristics and his/her athletic training history (surrounding gray frame) must be taken into account in every step, with special attention on those characteristics related with more marked RV remodeling (high training load and duration, dynamic exercise, older age group, male sex: RED FLAGS). Footprints represent how these features leave a mark that cannot be ignored. CMR, cardiac magnetic resonance; GLS, global longitudinal strain; LV, Left ventricle; NSVT, non‐sustained ventricular tachycardia; PVBs, premature ventricular beats; RV, right ventricle; RVFAC, RV fractional area change; TAPSE, tricuspid annulus systolic excursion; TDI, Tissue Doppler Imaging; TWI, T‐wave inversion

RV systolic function in athletes does not usually differ from nonathletes.^14^ However, in highly trained endurance athletes where the RV dilation is prominent, a slight reduction in RV global systolic function assessed by RV fractional area change may be shown.^28^ However, RV regional function is always preserved in athletes and the identification of regional wall motion abnormalities should be considered highly suspicious for ACM. Conventional echocardiography has lower relative sensitivity and specificity in differentiating physiology from ACM in athletes as compared to the general population. Thus, if any of these conventional parameters are abnormal, a comprehensive assessment of the RV becomes mandatory (Figure 3). Novel echocardiographic techniques have led to a more accurate assessment of cardiac mechanics providing a direct estimation of myocardial deformation. Their application facilitates the evaluation of RV function considering the complex geometry of this heart cavity. RV strain is predominantly limited to longitudinal deformation.^26^ In highly trained endurance athletes, some studies reported slightly reduced myocardial deformation RV values (assessed by speckle tracking echocardiography and tissue Doppler imaging) as compared to nonathletes, especially at the RV basal segment.^31^ However, in all cases, these mildly reduced values were within the normal range.^31^ A recent expert consensus document discussing ACM from the European Association of Cardiovascular Imaging has recommended the use of global RV strain in this setting, which has typically been demonstrated to be reduced in ACM patients and to be normal in athletes.^32^ A cutoff value for differentiating physiology from pathology of <20% (absolute values) has been proposed.^33^ In addition, the presence of mechanical dispersion of the RV contraction, which is typically not present in healthy subjects, has been proposed as an early sign of ACM^32^ and as an early predictor of future arrhythmic events.^34^ The use of deformation imaging techniques confirmed that ACM is not only confined to the RV. Even in patients with right‐dominant ACM phenotypes and their relatives, deformation imaging demonstrated a substantial incidence of LV involvement, especially at middle and apical regions of the LV posterolateral wall.^35^

Exercise echocardiography may be considered in athletes with prominent RV dilation and borderline or reduced systolic RV function in resting conditions. During exercise, healthy athletes are expected to effectively increase stroke volume and overall RV function, reflecting a good contractile reserve.^7^ In contrast, a blunted or insufficient increase of RV function in response to the exercise stimulus should raise suspicion of an underlying cardiomyopathy.^7^

### 4.2 Cardiovascular magnetic resonance (CMR)

Imaging allows an accurate and reproducible estimation of ventricular mass, volumes, and is considered the standard of reference for the evaluation of global and regional RV contractile function.^12^ Additionally, it provides tissue characterization. Normal values for ventricular size and function in white male athletes have been recently published in a meta‐analysis of studies involving CMR in apparently healthy competitive athletes from 18 to 55 years (Table 1).^15^ CMR studies in female athletes are scarce, but the data available confirmed what had been previously demonstrated in echocardiographic studies, that RV dimensions in women are lower as compared to men, independent of training status. Commonly, the RV volumes of well‐trained athletes (both absolute and indexed values) exceed the upper limits of the international guidelines and fulfill the minor volume criteria for ACM.^15^ Therefore, current European recommendations for the interpretation of cardiovascular imaging in the evaluation of athlete's heart^12^ established that, in the athletic population, RV should be considered enlarged only when the task force major volume criteria for ACM is fulfilled (≥110 mL/m^2^ for male and ≥100 mL/m^2^ for females).^12^, ^20^ As previously addressed, exercise training is expected to promote a harmonic enlargement of all cardiac chambers and thus, in healthy athletes, an enlargement of right heart chambers should be accompanied by parallel enlargements of left heart chambers. When facing an enlarged RV, rather than considering RV size values in isolation, the evaluation of the RV/LV dimensional ratios becomes essential. An RV/LV volume ratio greater than 1.2 assessed by CMR has been proposed as a cutoff to differentiate pathological and physiological biventricular remodeling in athletes.^12^

**Table 1:** Right ventricular morphologic and functional parameters assessed by 2D echocardiography and cardiac magnetic resonance in endurance athletes

2D‐echocardiography	D'Andrea, 2013^29^ (N = 395, 61% men)		Oxborough, 2012^28^ (N = 102, 85% men)		Sanz‐de la Garza, 2017^30^ (N = 40, 100% women)	
Dimensional parameters	Mean ± SD	URL	Mean ± SD	URL	Mean ± SD	URL
RVOT proximal (mm)	31.3± 6.3	43.9	34 ± 5	44	33.6 ± 2.9	39.4
IRVOT proximal (mm/m^2^)	17.0 ± 3.4	23.8	17 ± 3	23	21.8 ± 2.1	26.0
RVOT distal (mm)	27.3± 7.3	41.9	NA		NA	
RV basal diameter (mm)	38.1± 5.3	48.7	44 ± 5	54	36.5 ± 4.2	44.9
RV middle diameter (mm)	33.3± 5.4	44.1	NA		NA	
RV long diameter (mm)	80.1± 5.7	91.5	92 ± 9	110	61.8± 5.7	73.2
RVEDA (cm^2^)	NA		26 ± 5	36	18.6 ± 2.7	24.0
IRVEDA (cm^2^/m^2^)	NA		13 ± 2	17	11.6 ± 1.7	15.0

Functional parameters	Mean ± SD	LRL	Mean ± SD	LRL	Mean ± SD	LRL
TAPSE (mm)	23.7± 2.8	18.1	NA		NA	
RVFAC (%)	49.3± 3.7	41.9	47 ± 7	33	49.3 ± 5.1	39.1
RV TDIs' (cm/s)	14.8± 2.9^a^	9.0	11 ± 1.3^b^	8.4	9.8 ± 1.0^b^	7.8

Cardiac magnetic resonance	D'Ascenzi, 2018^15^ (N = 250, 100% men)		Domenech‐Ximenos, 2019^36^ (N = 44, 100% women)	
Dimensional Parameters	Mean ± SD	URL	Mean ± SD	URL
RVEDV (mL)	230 ± 7	244	145 ± 25	195
IRVEDV (mL)	120 ± 7	134	92 ± 16	124
RVESV (mL)	101 ± 4	109	69 ± 12	93
IRVESV (mL)	55 ± 2	59	42 ± 8	58
**Functional parameters**	**Mean ± SD**	**LRL**	**Mean ± SD**	**LRL**
RVEF (%)	54 ± 1	52	54 ± 4	46

Abbreviations: IRVESV, indexed right ventricular end‐systolic volume; IRVOT, indexed right ventricular outflow tract; LRL, lower reference limit; RV long diameter, right ventricular longitudinal diameter; RV TDIs', peak velocity tricuspid lateral annulus by Tissue Doppler Imaging. IRVED, indexed right ventricular end‐diastolic volume; RVEDA, right ventricular end‐diastolic area; RVEDV, right ventricular end‐diastolic volume; RVEF, right ventricular ejection fraction. NA, not available; RVFAC, right ventricular fractional area change; TAPSE, tricuspid annular plane systolic excursion; URL, upper reference limit.

a From pulse tissue Doppler imaging acquisition.

b From color tissue Doppler imaging acquisition.

In resting conditions, RV ejection fraction in highly trained athletes was shown to be slightly lower (52%) than in the general population^15^ but, in any case, within the normal range. In addition, healthy athletes always exhibit normal regional RV function, having to consider regional wall motion abnormalities as a sign of an underlying cardiomyopathy. Therefore, in regards to RV global systolic function assessment by CMR, both minor and major TFC for ACM should be considered when evaluating an athlete, specifically: regional motional abnormalities together with RV ejection fraction <45% as minor criteria and <40% as major criteria.^12^, ^20^ The evaluation of RV performance during exercise has been demonstrated to be also feasible by CMR, providing an accurate estimation of RV contractile reserve.^37^

*Late gadolinium enhancement (LGE)* imaging aids in detecting myocardial inflammation and fibrosis of the LV. Currently, it is also applied for RV analysis, although its interpretation is more challenging due to the thin RV wall. Recently, three‐dimensional LGE has been applied for RV fibrosis evaluation with promising results.^38^ This innovative technique may provide a more accurate RV tissue characterization in the near future. Recent studies reported higher prevalence of minor local fibrosis at the RV insertion points in highly trained athletes as compared to nonathletes.^4^, ^36^ However, the significance of this finding is still unclear and, for the time being, should not be considered a pathological sign. The application of LGE has shown that, in right‐dominant ACM patients, the involvement of the LV is earlier than was previously thought based on traditional imaging modalities.^39^ In addition, in left‐dominant forms of ACM, a positive LGE could be the first sign of the underling cardiomyopathy. The LGE pattern in ACM patients is characterized to be subepicardial or midwall and to involve the posterolateral and inferior LV walls.^40^

Table 1 summarizes the most important studies that provide mean values and the range for RV functional and structural parameters assessed by 2D‐echocardiography and CMR in athletes involved in endurance sports disciplines in which the more marked exercise‐induced RV remodeling is expected.

## 5 FURTHER ASSESSMENT

### 5.1 Ambulatory monitoring and exercise testing

It can be extremely difficult to determine the significance of palpitations, isolated premature ventricular beats (PVBs), or non‐sustained ventricular tachycardia (NSVT) in athletes coming from RV, particularly when an extensive morphological and functional evaluation of RV was considered as normal in an athlete. The most common ventricular arrhythmias seen in athletes are PVBs.^41^ Their occurrence has been shown to be independent of training‐related physiological left ventricular remodeling indices^42^ and to be sensitive to brief periods of deconditioning in trained athletes.^43^ Multiple PVBs (≥2 per 10 seconds ECG‐tracing). Ventricular couplets, triplets, and NSVT are uncommon findings in athletes and prompt further evaluation even in the absence of apparent structural RV disease.^19^ An ambulatory ECG monitoring and a maximal exercise stress test to assess PVB response to exercise, burden and pattern of the arrhythmia should be performed in these cases. A high PVB burden (≥2000 PVBs per day), uncommon PVB morphologies (left bundle branch block and intermediate/superior axis or complete RBBB), an increased PVB frequency or runs of NSVT during exercise could be signs of an underlying cardiac disease.^44^ Modern small leadless ECG monitoring systems are especially useful in athletes as they provide accurate ECG recording even during exercise training sessions and for long periods of time. Deconditioning‐induced reduction of PVBs during follow‐up ambulatory ECG monitoring is indicative of a benign condition.^43^ In addition to aforementioned usefulness of exercise ECG to assess PVBs behavior and other potentially exercise‐related arrhythmias, this test might help to unmask abnormal blood pressure responses or underlying ischemia.

### 5.2 Invasive assessment

When diagnosis is still uncertain, an invasive approach may be considered. However, electrophysiological testing in athletes sometimes reveals controversial results. Sustained ventricular tachyarrhythmias are rarely induced even when NSVT are present in the ECG monitoring, and thus their clinical interpretation is not clear.^44^ Electroanatomical mapping with guided RV biopsy has been reported in athletes, considering myocardial inflammation, fibrosis, and/or fatty infiltrates often of unknown significance.^45^ Given the invasive nature of both procedures and the challenge of interpreting the results, these procedures are not recommended as routine practice. There is a need for more studies investigating noninvasive techniques that could provide an accurate diagnosis in athletes with complex ventricular arrhythmias and no evident structural heart disease.

### 5.3 Genetic testing

The role of genetic testing has been gaining interest in recent decades. Although indications are the same for athletes and nonathletes, it may have additional utility in athletes by contributing to the early detection of genetic cardiac conditions that may increase the risk of experiencing life‐threatening arrhythmias and sudden cardiac death.^46^ This is particularly important when an early diagnosis can factor into decisions that may be life‐saving, such to reduce exercise training load in a patient with an initial/hidden for of ACM.^47^ The yield of any genetic screening should be expected to decrease when the clinical picture is less clear. It is important to emphasize that a clinical diagnosis cannot be excluded solely on the basis of a negative result of molecular screening since there is a substantial part of patients in which genetic abnormalities cannot be identified by the current available genetic screening.^46^

## 6 STEPWISE ALGORITHM WHEN ABNORMAL RV REMODELING IS SUSPECTED

The following algorithm (Figure 3) summarizes the proposed work‐up to evaluate a potential pathologic RV remodeling in athletes. This task does not rely on a single parameter, measurement, or technique, but requires a comprehensive and integrative approach. Whatever the starting point for raising suspicion of abnormal RV remodeling (athlete's history, symptoms, ECG, cardiac imaging, or even in asymptomatic individuals during pre‐participation screening), it is useful to follow a stepwise approach from initial ECG to additional and further explorations on sequential steps of investigation.

Clinical traits are the framework where any parameter should be acknowledged and include training load (especially when dynamic disciplines are considered), male gender, and increasing age. ECG is considered the first supplementary exam to perform in pre‐participation screening in athletes. Second step aims to assess with echocardiographic measurements if RV dimensions exceed values expected in the athlete's population. If so (third step), functional RV parameters and the ratio LV/RV should be further evaluated. When any of these functional parameters are under normal limits and/or RV dimensions exceed the upper limits provided for the athlete's population, an underlying disease should be excluded. In inconclusive cases, further assessment (step 4) is required. Exercise ECG might help in the assessment of PVBs, exercise‐induced arrhythmias, abnormal blood pressure responses as well as in the detection of repolarization changes suggestive of ischemia or an underlying disease. An impaired RV contractile reserve, a significant decrease of RV global function and/or the presence of wall motion abnormalities (by CMR and/or exercise echocardiography) as well as positive LGE (CMR) should be considered pathological signs.
